# Small Pox and Vaccination

**Published:** 1862-07

**Authors:** H. Gibbons


### SMALL POX AND VACCINATION.

By H. GIBBONS, M. D.

At the present juncture, when there exists an epidemic condition favorable to the development of variolous disease, it is important, both to the profession and the public, that our knowledge of the means of prevention, by vaccination and revaccination, be brought out and spread abroad. The following general conclusions may be regarded as tolerably well established by observation and experience:

In many persons a single vaccination is an effectual protection from Small Pox through life, whilst in others it appears to "wear out," to a greater or less extent.

Persons are very seldom attacked with Small Pox within five years after vaccination.

The age most liable to the disease is from 15 to 30 or 35 years.

When an epidemic influence favorable to Small Pox prevails, one person at this age out of every four or five exposed to the infection, will take the disease.

While Small Pox in the unprotected is fatal in at least one-fourth the cases, it is so modified after vaccination as to cause death in only one case out of fifty, or perhaps a hundred.

Inoculation is a surer protection than vaccination; but persons taking the disease after inoculation have it more violently.

Vaccination will not take a second time, to produce a *genuine* vesicle, in more than one case out of 80 or 100. And even then the vesicle is apt to be small and the scab thin.

Revaccination will always produce *some effect*, if the matter be recent and genuine. The arm may itch for a day or two, and the irritation vanish; or it may proceed to swell and fester, remaining sore for several days; or it may progress still further, causing much inflammation and often fever, being as severe as the genuine vaccination, and even more so.

The effect of revaccination depends much on the manner of performing the operation. If the virus be tucked into the skin with two or three small punctures, it will not cause much of a sore, unless the system be susceptible, in some degree, to the true cow pock. But if the skin be extensively scratched or abraded and the virus applied, it will nearly always make a severe sore, which may be nothing more, however, than a poisoned wound.

If the vaccination inflames and becomes sore in less than a week, it is spurious.

Though revaccination produces nothing more than spurious, irregular and imperfect vesicles or pustules, yet it appears to act on the system so as to protect it from the Small Pox.

When the Small Pox makes its appearance in a city or neighborhood, and shows a tendency to spread, every person, between 15 and 40, who is exposed in the street or in his business, should be revaccinated. This epidemic character of the disease is not apt to occur more frequently than once in five or six years.

When an individual is seized with Small Pox, every person, old or young, residing in the house, or frequenting it, should be immediately vaccinated. This has been my invariable practice—in Wilmington, Delaware, where the disease prevailed, extensively, in 1828; in Philadelphia, where it was slightly epidemic, I think in 1847; and in San Francisco, where it prevailed about nine years ago; as well as at other times, when occasional cases occurred. *I have never known a single instance* of the disease spreading or being contracted by a second member of the family, when this precaution was adopted.

The production of a sore arm by vaccination is no proof that the individual could have taken the Small Pox. Persons over forty years of age, who are generally secure from this disease, will take the revaccination in the same manner as those who are younger.

Some individuals will never take the vaccine disease, though the operation be repeated year after year. They possess a natural exemption. Experience shows that these persons are also exempt from Small Pox.

It often happens that children will not take the vaccination at one time, owing to some peculiar condition of the system. But, in a few months, a change takes place, and they become susceptible of the disease. If it be repeated, year after year, without success, the individual is regarded as exempt for life, both from the cow pox and the small pox. These natural exempts may be about one in a thousand in the population. I have lately vaccinated two individuals, both of them heads of families and near the age of thirty, who had been vaccinated, in past years, again and again, without effect, and yet who took the cow pock in its genuine form in my hands. These were, to me, anomalous cases. One of them, a lady, had nursed persons with the Small Pox, and resisted the infection. It is quite probable they would both have taken the disease at the present time, if exposed to it.

I am aware that some physicians consider the genuine cow pock to result from revaccination in a much larger proportion of cases than above stated. But I think a rigid inspection of the arm will show that the sore is wanting in some of the characteristics of the true kine pock. It may be a pustule instead of a vesicle—it may lack the central depression—it may not present the *regular* areola. The sore may approach very nearly to the genuine disease, and yet be imperfect or spurious.—*San Francisco Med. Press.*

